# The Impact of Living with Parkinson's Disease: Balancing within a Web of Needs and Demands

**DOI:** 10.1155/2018/4598651

**Published:** 2018-07-29

**Authors:** Catharina Sjödahl Hammarlund, Albert Westergren, Ingrid Åström, Anna-Karin Edberg, Peter Hagell

**Affiliations:** ^1^The PRO-CARE Group, Faculty of Health Science, Kristianstad University, Kristianstad, Sweden; ^2^Department of Health Sciences, Lund University, Lund, Sweden; ^3^The Research Platform for Collaboration for Health, Faculty of Health Science, Kristianstad University, Kristianstad, Sweden

## Abstract

This study explores the impact of living with Parkinson's disease (PD). Nineteen persons (11 women) aged 55–84 diagnosed with PD 3–27 years ago participated. Data were collected through semistructured interviews, which were recorded, transcribed verbatim, and analysed by qualitative content analysis. Four categories represented the impact of living with PD: “Changed prerequisites for managing day-to-day demands,” “Loss of identity and dignity,” “Compromised social participation,” and “The use of practical and psychological strategies.” There was a shift from an internal to an external locus of control in managing, control, competence, relatedness, and autonomy. According to self-determination theory, a shift towards extrinsically motivated behaviours may occur when these basic needs are thwarted, leading to compensatory strategies or needs substitutes with negative consequences on health and well-being. We suggest a needs-based approach as an important starting point to better understand the consequences of living with PD and to explore the means for people with PD to acquire an improved quality of life on their own terms. In conclusion, our findings suggest for a shift in focus, from a biomedical to a needs-based approach to understand the impact of living with PD and facilitate more person-centred care and person-centred outcome measurement.

## 1. Introduction

Parkinson's disease (PD) is a progressive, neurological disease involving motor (e.g., bradykinesia, tremor, rigidity, and postural impairment) and nonmotor (e.g., depression, anxiety, sleep disorders, fatigue, dysautonomia, and pain) symptoms [1, 2]. Its symptom profile and progression differ between individuals. The core pathology believed to cause the main motor symptoms is a striatal dopamine deficit due to progressive loss of nigrostriatal dopaminergic neurons [2]. Symptomatic dopaminergic therapy is initially successful, but a fluctuating drug response and dyskinesias often develop after some years. With the occurrence and progression of both motor and nonmotor symptoms, often in complex and fluctuating patterns [1, 3], the disease is typically perceived as unpredictable and difficult to control [4].

An increasing number of studies have considered the consequences of PD characterized symptomatic profiles, relationships among symptoms, and the impact of various features of the disease from the perspective of persons living with PD [5–7]. Previous studies have reported how persons with PD (PwPD) experience various disease aspects, such as loss of motor control, walking difficulties, environmental influences, cognitive and executive dysfunctions, emotional reactions, sleep disorders, difficulties in managing activities of daily living, neuropsychiatric reactions, social withdrawal and isolation, communication difficulties, and loss of physical and psychosocial competence [8–13]. Collectively, these and other studies have provided important insights into several aspects of PD. However, available studies have typically focused on relatively specific aspects of the disease (e.g., communication, fatigue, and walking) or subgroups of PwPD, such as women and middle-aged or older people [14–17]. Few qualitative studies have attempted to capture the overall impact of PD on daily life, and available studies [18, 19] stem from the era before the introduction of, for example, modern dopamine agonists and enzyme inhibitors [20]. To the best of our knowledge, there are no studies that take a holistic approach when describing the consequences of living with PD. The aim of this study was therefore to explore the overall impact of living with PD.

## 2. Materials and Methods

### 2.1. Participants and Recruitment

Thirty consecutive Swedish-speaking people with clinically diagnosed PD from a Swedish movement disorder clinic were selected by purposeful sampling and invited to participate [21]. Specifically, we aimed to achieve variations regarding age, gender, disease duration, and PD severity. Exclusion criteria were ongoing psychiatric side effects from medication and clinically significant comorbidities that could compromise the ability to participate (e.g., dementia), as determined by a specialized PD nurse. Nineteen persons, 11 women and 8 men aged 55–84 (median: 66 years, q1–q3: 61–73 years), were able to participate and provided signed informed consent. They were diagnosed with PD since 3–27 years (median: 11 years, q1–q3: 7–14 years). Disease severity ranged from mild bilateral disease (Hoehn & Yahr stage II) to severe PD with inability to stand or walk unless aided (Hoehn & Yahr stage V) [22]. All participants were treated with levodopa, and additional antiparkinsonian therapy consisted of oral dopamine agonists (pramipexole and ropinirole), COMT inhibitors (entacapone and tolcapone), amantadine, subcutaneous apomorphine infusion, and deep brain stimulation. The study was conducted in accordance with the Declaration of Helsinki and was approved by the local research ethics committee.

### 2.2. Data Collection

Data were collected by means of face-to-face semistructured interviews, which were recorded and transcribed verbatim. The interviews lasted for 60 to 90 minutes and followed a preprepared interview guide. All interviews were conducted face to face in privacy, without the involvement of caregivers/family members. The interviewer started each interview with the phrase: “We are interested in learning more about how you have experienced the consequences of Parkinson's disease, now and in the past. Could you please tell me how you first noticed the disease?,” followed by “Can you describe how the disease has influenced your daily life?.” The interview guide consisted of a checklist of areas that might potentially add to the understanding of the consequences of PD. The areas were: at home, at work, in personal relationships and social life, cognitive ability, personal care, leisure activities, sleep, and rest. If not covered spontaneously by the respondent, these areas were explored by open-ended questions. When needed, prompts were used to encourage respondents to explore and expand statements in greater depth.

### 2.3. Data Analysis

Following verbatim interview transcription, data were analysed as described by Graneheim and Lundman [23] and Patton [21], according to the following steps: (1) All interviews were read separately several times in order to get an understanding of the essence of each interview and to identify primary patterns in the data. (2) Meaning units were identified and (3) coded with regard to the content. (4) Clusters of aspects that emerged in the coded data were organised in subcategories. (5) Subcategories were sorted by their conceptual representation, ending up in four main categories, representing a more abstract level of understanding. To assist and organise the analysis, the OpenCode 4.0 software for qualitative analysis was used. OpenCode 4.0 is a software for coding and categorising qualitative interview or observational data. The program was originally developed for grounded theory and qualitative content analysis, but can be used with most qualitative methods [24].

## 3. Results

The impact of living with PD can be understood as “Changed prerequisites for managing day-to-day demands,” “Loss of identity and dignity,” “Compromised social participation,” and “The use of practical and psychological strategies” with internal variations seen as subcategories (Table 1).

### 3.1. Changed Prerequisites for Managing Day-to-Day Demands

The participants described how prerequisites for managing day-to-day demands had changed due to deteriorating physical, psychological, and cognitive functioning. This change affected most activities, which became difficult to manage, as some perceived their abilities to be severely affected or completely lost. The changed prerequisites concerned deteriorating physical functioning, fatigue, psychological changes, and mood swings as well as cognitive impairments.

*Deteriorating physical functioning* was evident in that the participants described that they had become slower no matter what they were doing and that everything took more time. Walking was more difficult because they felt stiff and it was increasingly hard to move the legs and feet. Walking pace had slowed down and keeping balance was more difficult. Fear of falling was common, and many had experienced several falls. They described that they sometimes suddenly could not move at all and felt like a statue, and there was nothing they could do about it.

*I couldn't walk any further than maybe out of the house and into the garden … then I found myself in a world that was totally my own … . I became completely stiff … my wife shouted at me that “you have to move”, but I was completely stuck … .* (Participant 8)

Other physical changes included painful cramps and pain in general. In combination with deteriorating fine motor abilities, daily activities (e.g., chores, shopping, getting dressed/undressed, and taking care of personal hygiene) became time-consuming, difficult to perform, and physically and psychologically challenging.

*It takes time to do the buttons … . It's irritating sometimes. It takes a long time before you have finished dressing… *. (Participant 10)

The participants described *fatigue* as an overwhelming and general feeling of tiredness and loss of energy. A sense of feeling completely worn-out and unable to take action could appear suddenly and at any time. Most of the time nothing could be done to solve the situation except to go to bed. Fatigue was also described as being both physical and psychological.

*I become so … utterly worn out. I can't hang my clothes… , put the dishes away. I can barely find the strength to go to the bathroom, or brush my teeth, because nothing works, you see. I just get slow, indifferent … . I don't care about anything but to go to bed … and just lie down…* . (Participant 1)

*Psychological changes and mood swings* were typically perceived as being related to physical deterioration and were described as difficult to control, for example, a sense of panic and powerlessness. Participants worried about the future and about what would happen when they could no longer care for themselves. Thoughts like these brought about anxiety and fear. Some felt depressed and downhearted most of the day, sad at being disabled, alone, and unable to do what they wanted to do. These thoughts could become destructive and murky, and some had feelings of wanting to die; what was the point of staying alive when life itself offered so little?

* … maybe … . I need help to look back on my life … Well to … to be able to see the meaning of life … to shed some light over this darkness … . To go on living or not … there are … there are days when I feel that everything is really bad and … there is no point in anything …* . (Participant 15)

Participants described how *cognitive impairments* were manifested, for example, problems in remembering and concentrating. Those who used to read a lot found it difficult to remember what they just read and to understand the context. Communicating with others was difficult as names were often forgotten, and what to say and finding the right words became even worse when they were nervous. It became difficult to do things that they used to do, for example, cooking or carpentry as the ability to count and measure was compromised. They described problems organising and keeping papers and other things in order; it felt like the brain was in a state of chaos.

*It's just like I … . I become indifferent … to some things. Very hard to keep things in order … *[laughs]. *I move things from one place to the other and I think that I have put everything right and then everything is … . There's some … there's some disorder … some chaos in the brain… *. (Participant 1)

### 3.2. Loss of Identity and Dignity

As the disease progressed, dependency on help with practical matters increased and family relationships changed. Participants expressed bitterness and shame of the person they had become and tried to conceal the disease. They noticed that the physical and mental changes affected their self-esteem, as well as how they were perceived by others. They felt humiliated and less confident when judged by others. Loss of identity and dignity altered family relationships, gave a sense of lost identity, and a sense of being worthless.

*Altered family relationships* involved affected the bond between husband and wife. Some described that their partner was afraid of future demands and responsibilities and was away more often. As the illness progressed, some families decided to redistribute household and family responsibilities. They found it difficult to ask for help as they wanted their families to treat them as they used to, without showing any special considerations. Participants became more dependent on practical help from their family members, for example, cooking and other chores, but also their personal hygiene and getting dressed, as well as concerns regarding their personal safety.

*Well, I think that it feels very bad … needing to ask for help … to get dressed … and again when I want to go to bed … and at the same time one becomes really frightened … if I was alone in the house, what was I supposed to do … and imagining things like … . There is nobody here, what if I were to fall … .* (Participant 8)

*A sense of lost identity* was described by all participants. Although they felt that deep inside they were still the same person, they were also aware that they were physically changing. The posture became more stooped, their faces looked more tired, and they were not as fast and skilled as they once were. They could have held positions in management and family responsibilities, a person that others would seek advice from and see up to. Now, they had to give up doing things or taking responsibilities, both at home and at work. They found themselves being dependent on others. This made them feel belittled, insecure, and less confident, as if they no longer belonged to this world. They felt themselves to be a professional and a personal failure, something that deprived them of their self-image and identity.

*… well, then you become sort of powerless … and indecisive … . You can't decide … you can't make up your mind… .* (Participant 5)

*A sense of being deprived of one's self-worth* could appear when meeting with others. It made them feel uncomfortable and resented if they felt that someone pitied and felt sorry for them. They wanted to be treated as a valid citizen although they had PD, just like they had been treated before the diagnosis. Some also worried that they would be taken for an alcoholic due to physical symptoms such as balance problems and tremor. They noticed that people sometimes stared or looked at them in an odd way, and they also felt exposed to naive and hurtful comments.

*Although I have Parkinson's disease, I want to be respected. I want to be able to do … take care of things like I always did in the past and I want to take an active part in society … hmmm … and I don't want to be looked down upon in any way … I want to be accepted even though I do have Parkinson's.* (Participant 15)

### 3.3. Compromised Social Participation

The prerequisites for taking part in social activities had changed and affected all aspects of their social life. They felt increasingly socially isolated, which in part was due to feeling embarrassed of their changed persona. In addition, some had speech problems, which made communicating difficult. Compromised social participation had to do with limited ability to meet with others, feeling socially isolated, social embarrassment, and speech problems.

*Limited ability to meet with others* and decreased activities in daily life were common. Not being able to drive was problematic as it made it more difficult to get around. Using public transportation required planning and was regarded as inconvenient. Other activities that were affected were holding cards whilst playing bridge, outings in the countryside, riding, playing football, or going out dancing. It was also difficult to manage stairs, to go in and out of shops, do the shopping, and other daily activities.

*And when one needs to go into shops, which rarely happens, it's not easy when there is a flight of stairs that has to be managed.* (Participant 10)

*Feeling socially isolated* and being alone was hard. Even if it sometimes was preferable to be alone, there was also a feeling of being avoided, even by close friends. Participants often wished something would happen to break the silence of their secluded life, for example, a phone call or a letter. Even if there was only a bill it was read thoroughly as a strategy to feel connected to the outer world.

*… well, from the moment I have the letter in my hand, I open it and it … it … just by doing that, it's something that makes you feel more alive … because it's a moment that feels exciting … . What's inside the envelope, what does the letter say … and then you start reading it … and if it's a bill I start to take an interest to it … you make as much of it as possible … in order to … live on… *. (Participant 9)

The participants described *social embarrassments* as their facial expressions and nonverbal communication were reduced. Involuntary movements were a problem, for example, when having dinner with family and friends. Glasses and cups could suddenly be knocked over, tremors made it difficult to use cutlery and handling food or drink. Eating was time-consuming, and they felt embarrassed by keeping others waiting. They felt bitter at having the disease, which made them try to hide their symptoms.

*It was like … when I had customers … and they sort of … why do you walk so funny? And then I  … oh, it's just because one of my toes is hurting … or something like that … hmm … that's how I kept it hidden for … well … at least … certainly for ten years… .* (Participant 8)

*Speech problems* were frustrating to the point where some stopped talking altogether, and others found it difficult to articulate. Being nervous or stressed tended to make it worse. These problems could make it impossible to speak on the phone, and consequently people stopped calling.

* … they told my children … to tell me that … that … we … we … we … don't understand what he's saying so there is no point calling … . And I understand that … . I find it equally … equally embarrassing… .* (Participant 9)

### 3.4. The Use of Practical and Psychological Strategies

The participants used different strategies to adapt to various situations. Trying to find practical solutions were most common, for example, seeking information, planning, and using aids. Other examples of strategies used to promote psychological well-being were to have a positive attitude or to compare one's own situation to that of others. Practical and psychological strategies used were to be able to foresee and plan, to compensate for lost functioning, trying to maintain a positive attitude, using downward comparisons, and accepting support from health care.

*To be foreseeing and plan* was necessary when managing everyday life. Most things took more time, and those with cognitive difficulties needed detailed planning, taking notes, and doing things step-by-step. Medication required a great deal of planning and most participants followed a schedule. Even though all precautions were taken, it was still uncertain if they would manage to go through with their plans due to the unpredictability of the disease. They felt embarrassed when they had to cancel at the last minute.

* … I don't always make plans just for myself. Sometimes there are others involved and it feels really bad to always be the one who cancels … for example if we're going to the movies or something… .* (Participant 5)

*Various compensatory strategies were applied* to compensate for lost functioning, for example, doing chores sitting down instead of standing up or to avoid reaching for things. Several situations were avoided altogether to minimise the risk of falling and being injured, for example, to shower instead of taking a bath. Some also chose to pay for the services that they were unable to do, for example, cooking and cleaning.

*Well, I … eh … I … eh … do the dishes, when I've finished eating … for other chores like cleaning up, I've help from others … hired help … and my children they cook … when they cook for themselves, they also cook for me and then they freeze the food and bring it to me. I can take care of my personal hygiene. I take a shower every morning, but … eh … to take a bath, which I used to love doing … it's not possible anymore. I can't get out of the bathtub.* (Participant 9)

*Trying to maintain a positive attitude* was used to feel psychologically better, for example, trying to be optimistic, not to worry, enjoying the moment, making the best of the situation, trying to live life as normally as possible, and pushing all thoughts of the future aside. PD could progress in so many ways that they found it best to settle for what little they had and make the most of every moment. Some described the importance of fighting back and not giving up. For example, thinking of children and grandchildren was also helpful in finding enough strength to keep on living.

*I have to start from how it is … and make the best of the situation … and so far, that's worked. It's not really a disaster to be ill … . Because you become sort of … you look at life differently and become more grateful… .* (Participant 14)

The participants were *using downward comparisons*, comparing themselves with those worse off, for example, while attending support group meetings. This strategy was helpful in realising that they still functioned relatively well, given the circumstances. However, others described the opposite reaction as it could make them worried and anxious that they might end up in a similar situation.

*Well, of course you compare yourself with others … . You make certain comparisons with … well, if you are sitting down … having a conversation with someone, you tell yourself … well, the things that he's doing aren't something that I do … you sort of compare yourself with that person … and then you choose to look at the best parts of the situation, and I tell myself that I'm much better off than he is… .* (Participant 8)

The participants also used self-deception, that is, convincing themselves that things were not as bad as they might seem. Regardless of the individual's specific strategy, it was about finding a way that worked, to adapt and try to accept the situation.

*Accepting healthcare support* was important in managing the situation, even if accessibility was an issue. They wished that it would be possible to have one person to turn to for support and felt that the time between scheduled clinic visits was too long.

*So she* [the physician] *really helps me, you know … and I feel that she's very supportive … like … she's always happy and kind when I call her and … never … never sort of stressed and she listens to what you have to say and … so  … . I think that  … that's very positive … .* (Participant 1)

To use various kinds of aids was important, such as alarm devices to remind them when to take their medicines or a special chair to use in the shower. To be able to manoeuvre a wheelchair with a remote control improved independence. Some also found the use of a cane to be supportive, whereas others could consider walking aids as stigmatizing. However, using some of these aids, for example, a walker could be difficult in bad weather, such as snow or if there were stairs.

## 4. Discussion

This study explored the overall impact of living with PD from the perspective of PwPD. The results showed that living with PD incorporated a complex web of changed prerequisites for managing day-to-day demands and social participation, which in turn contributed to a perceived loss of identity and dignity. Living with PD also involved developing practical and psychological strategies to cope with the situation. These results may be thought of as a delicate balance within a web of needs and demands.

Psychological symptoms and mood swings were intertwined with cognitive and physical problems, adding to the struggle of managing the demands of everyday life. The participants felt depressed, low-spirited, and were worried about the future. Some held dark and destructive thoughts and considered intentionally ending their lives. In a previous study, suicidal and death ideation was present among one-third of persons with PD [25]. Depression is one of the most common nonmotor symptoms in PD [1] and has considerable impact on health-related quality of life [5, 6]. The physical changes also affected appearance. To look different provoked a sense of vulnerability due to feeling judged and evaluated by strangers. Those who suffered from tremor or balance impairments could be thought of as being intoxicated or worse. In agreement with earlier studies [17, 26, 27], this was found embarrassing and made participants feel deprived of their self-worth and shameful as they could no longer live up to social norms.

**Figure 1:**
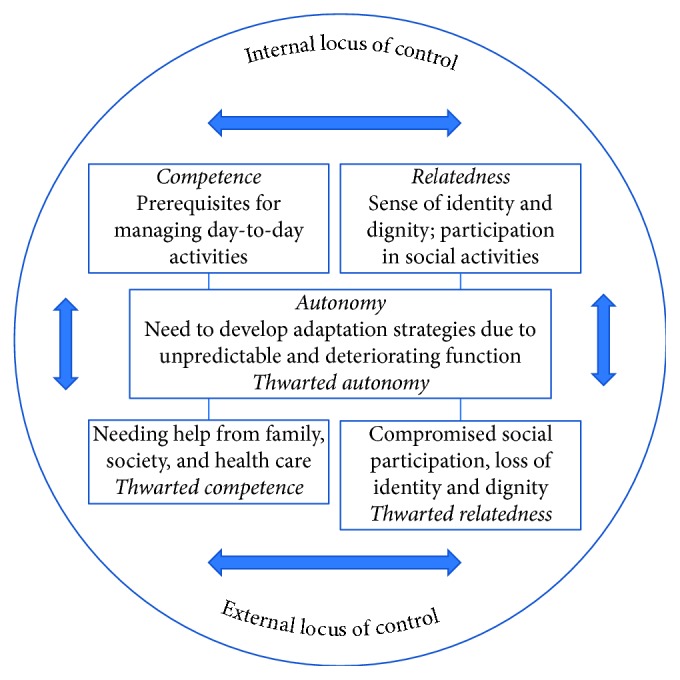
Balancing within a web of needs and demands. A model based on self-determination theory of how people with Parkinson's disease strive to manage the continuous shift between internal and external locus of control, in attempting to maintain competence, relatedness, and autonomy.

The results can be interpreted as a shift from an internal towards an external locus of control, in order to manage their sense of lost control and unpredictability, as the disease progressed (Figure 1). According to self-determination theory (SDT) [28, 29], this shift and the described distress and helplessness may be the result of thwarted basic needs. SDT states that three basic psychological needs are necessary conditions for people's well-being and health: *competence* (a sense of confidence and feeling effective in interacting with the surrounding society), *relatedness* (belongingness, feeling connected to others) and *autonomy* (acting from interest and integrated values). When these needs become thwarted, a shift towards extrinsically motivated behaviours may occur. Such behaviours, for example, to no longer feel capable of changing one's situation (thwarted competence and autonomy) or feeling sad to be alone (thwarted relatedness), may lead to compensatory strategies or substitutes that will not benefit health and well-being. Interestingly, shifting locus of control and thwarted basic needs have been found to predict poor psychological well-being, depression, and ill health [28, 29]. SDT may thus help provide a deeper understanding of the complexity of the impact of living with PD, as the social conditions and other aspects that needed to be managed become more challenging as the disease progressed.

The vast range and complexity of PD-related symptoms and problems, and their impact on participants' needs and demands may seem overwhelming. Nevertheless, rather than a separable multidimensional array of disease manifestations, symptoms, and problems, these and other aspects are intermingled and appear to form a unity where all parts are facets of a single dimension of the impact of living with PD. This is similar to the findings in a previous study where PwPD and health care professionals (HCPs) conceptually mapped out the interrelationships among aspects deemed important to consider when evaluating interventional outcomes in PD [30]. While HCPs tended to group aspects into clinically distinct areas, PwPD mapped out a more intermingled pattern. This finding was strengthened in the present study, suggesting that the impact of PD from a patient perspective may be described as unidimensional but heterogeneous. Moreover, the impact as described here was largely expressed in terms of unmet needs that change over time, followed by different adaptation strategies. This has implications in terms of person-centred outcome measurement and supports a needs-based quality of life model [31–33]. This suggests that the impact of PD as well as needs-based outcomes may be interpreted in relation to SDT.

The changed ability to take part in various social activities restricted interactions with family, friends, and others. These aspects had consequences for the psychological and emotional aspects of reduced self-worth and the social identity. To facilitate meeting the needs and demands of everyday life, problem-focused and emotion-focused strategies were used to reduce stress and worry and to facilitate adaptation in various situations [34, 35]. However, for some, it was not always realistic to expect that everything would go according to plan. To be able to foresee possible problems and to plan ahead gave the participants a sense of control over the situation, but also reduced the possibility of acting spontaneously [16].

To maintain a positive attitude was one emotion-focused strategy that facilitated emotional balance and well-being. Some found new acquaintances in new contexts, for example, in a PD association, where they met others in similar situations. However, although some were comforted by being able to help those who were worse off, it could also be distressing for those who were afraid of ending up in a similar situation, which has been reported previously [36, 37].

The physical challenges and perceived distress in managing everyday activities and participating in society appear to be similar across different brain disorders [38]. It may thus well be that two persons with different disorders can have more in common than two persons with the same diagnosis, as their challenges not only relate to their health conditions. Therefore, the experienced impact of various disorders may be better represented by use of a needs-based rather than a function or symptom-based approach [31–33]. Arguably, the needs-based approach may also be beneficial from a clinical perspective, by being better positioned to facilitate a more person-centred care. The needs-based quality of life model postulates that life gains its quality from the ability and capacity of the individual to satisfy his/her needs [31–33]. This is in general agreement with the theory and empirical experiences of SDT [28, 29]. The results of our study point to the need for a shift from a biomedical to a more needs-based approach to better understand the impact of living with PD as well as interventional outcomes from the patient's perspective and to facilitate a more person-centred care.

Our findings illustrate the value of qualitative inquiry in order to gain a deeper knowledge and understanding of the impact of long-term illness from a patient perspective. Such information may also lay the foundations for novel and more person-centred outcome measures. It is well established that in addition to being conceptually and theoretically based, the content of any outcome measure intended to represent the patients' perspective should be derived directly from patients with experience from the target disorder [38, 39]. However, these two fundamental components do not appear to have been widely integrated in the development patient-reported outcome measures for PD [40–45].

## 5. Conclusion

We have found the impact of living with PD to be a complex unity of intermingled symptoms and problems that entail a balance within a web of needs and demands. Seen from this perspective, we suggest that a needs-based approach represents an important starting point to better understanding the consequences of living with PD and to explore the means for people with PD to acquire an improved quality of life on their own terms. This has important implications for understanding living with PD, as well as for designing improved and more person-centred interventions and outcome measures.

## Figures and Tables

**Table 1:** The impact of living with Parkinson's disease.

Categories	Subcategories
Changed prerequisites for managing day-to-day demands	*Deteriorating physical functioning* *Fatigue* *Psychological changes and mood swings* *Cognitive impairments*

Loss of identity and dignity	*Altered family relationships* *A sense of lost identity* *A sense of being deprived of one's self-worth*

Compromised social participation	*Limited ability to meet with others* *Feeling socially isolated* *Social embarrassments* *Speech problems*

The use of practical and psychological strategies	*To be foreseeing and plan* *Various compensatory strategies were applied* *Trying to maintain a positive attitude* *Using downward comparisons* *Accepting healthcare support*

## Data Availability

Anonymized original data may be obtained at the discretion of the corresponding author upon request.
